# Prognostic Relevance of Thyroid-Hormone-Associated Proteins in Adenoid Cystic Carcinoma of the Head and Neck

**DOI:** 10.3390/jpm11121352

**Published:** 2021-12-12

**Authors:** Julia Schnoell, Ulana Kotowski, Bernhard J. Jank, Stefan Stoiber, Elisabeth Gurnhofer, Michaela Schlederer, Gregor Heiduschka, Lukas Kenner, Lorenz Kadletz-Wanke

**Affiliations:** 1 Department of Otorhinolaryngology, Head and Neck Surgery, Medical University of Vienna, 1090 Vienna, Austria; julia.schnoell@meduniwien.ac.at (J.S.); ulana.kotowski@meduniwien.ac.at (U.K.); bernhard.jank@meduniwien.ac.at (B.J.J.); lorenz.kadeltz@meduniwien.ac.at (L.K.-W.); 2 Department of Pathology, Comprehensive Cancer Center, Institute of Cancer Research, Medical University of Vienna, 1090 Vienna, Austria; stefan.stoiber@meduniwien.ac.at (S.S.); elisabeth.gurnhofer@meduniwien.ac.at (E.G.); Michaela.Schlederer@meduniwien.ac.at (M.S.); 3 Christian Doppler Laboratory for Applied Metabolomics, 1090 Vienna, Austria; 4 Unit of Laboratory Animal Pathology, University of Veterinary Medicine, 1210 Vienna, Austria; 5 CBmed GmbH-Center for Biomarker Research in Medicine, 8010 Graz, Austria

**Keywords:** NIS, CRYM, THRB, adenoid cystic carcinoma, prognosis

## Abstract

The proteins sodium iodide symporter (NIS), μ-crystallin (CRYM), and thyroid hormone receptor beta (THRB) have been associated with prognosis in various cancer entities. While NIS and THRB may serve as possible therapeutic targets, the role of CRYM in cancer is still unclear. Protein levels of 44 patients with adenoid cystic carcinoma of the head and neck were analyzed using immunohistochemistry and correlated with clinicopathological data and outcome. NIS was positive in 72%, CRYM was positive in 55%, and THRB was positive in 39% of the patients. CRYM-positive adenoid cystic carcinomas were associated with a better cause-specific survival. Thus, our data indicate that CRYM might be a suitable positive prognostic marker in adenoid cystic carcinoma of the head and neck. Furthermore, expression of NIS was present in most patients and therefore evaluation of the use of radioiodine treatment is recommended.

## 1. Introduction

Adenoid cystic carcinoma (ACC) of the head and neck is one of the most common salivary gland carcinomas, with an incidence of between 1.2 and 4.5 cases/million per year. ACC is characterized by perineural invasion, distant metastasis (into lung, bone, or liver), and late recurrences. The mainstay of treatment is complete surgical resection and postoperative radiotherapy in the case of advanced disease. Systemic therapy is reserved for progressive disease and palliation, due to its low response rates [1,2]. Although the short-term cause-specific survival (CSS) rate is rather favorable (84% after 5 years), it continuously drops over time, to 72–47% after 10–30 years [3]. Poor prognostic factors are advanced tumor stage, perineural invasion, and solid histologic patterns [1,2,3]. In other cancer entities, thyroid hormones and associated proteins are associated with tumor promotion or suppression but have so far not been investigated in the context of ACC [4]. Therefore, we investigated the protein levels of sodium iodide symporter (NIS), μ-crystallin (CRYM), and thyroid hormone receptor beta (THRB) in ACC of the head and neck.

To produce thyroid hormone, active transportation of iodide into thyroid cells is mediated by NIS, also known as Solute Carrier Family 5 Member 5 (SLC5A5). In thyroid cancer, this mechanism is exploited by the use of radioiodine, one of the main treatment options [5]. Since the side effects of radioiodine treatment commonly include sialadenitis and later xerostomia, we hypothesize that NIS might be a promising therapeutic target in ACC [6,7]. Furthermore, Gainor et al. showed that the NIS protein is present in the majority of ACC patients. However, the prognostic value was not investigated [8]. In other cancer entities, high levels of NIS are associated with a worse prognosis [9,10,11,12].

In general, thyroid hormones regulate cell metabolism, growth, and development [4]. CRYM, also known as NADP-regulated thyroid hormone binding protein (THBP), binds triiodothyronine (T3) and thus increases the cytoplasmatic concentration of T3. To exhibit its nuclear effect, T3 is released from CRYM via dissociation of NADPH [13]. To date, the role of CRYM in cancer has only been studied in prostate cancer. Here, CRYM antagonizes thyroid hormone signaling and patients with high levels of CRYM show a better prognosis [14].

In the nucleus, thyroid hormones regulate transcription via thyroid hormone receptors. Although an abundance of thyroid hormones is commonly linked to tumor promotion, the nuclear-ligand-dependent receptor THRB is associated with tumor suppression. THRB mediates apoptosis and suppression of metastasis and thus shows promising therapeutic potential [4,15,16,17]. Furthermore, high levels of THRB are associated with a better outcome in breast cancer [18,19].

The aim of this retrospective study was to investigate protein levels of the thyroid-hormone-associated proteins NIS, CRYM, and THRB in patients with ACC of the head and neck and their correlation with prognosis.

## 2. Materials and Methods

### 2.1. Patients and Study Design

In this retrospective, single-center, cohort study, 44 patients were included who were treated for ACC of the head and neck area between 1996 and 2016 at the Department of Otorhinolaryngology and Head and Neck Surgery of the Medical University of Vienna. Patients were excluded if they had a second carcinoma, were under 18 years old, or had incomplete records. Data were collected by medical chart review. The study was approved by the ethics committee of the Medical University of Vienna (EK 1517/2018). 

### 2.2. Tissue Microarray and Immunohistochemistry

A tissue microarray was constructed, and immunohistochemical staining was performed on the paraffin-embedded samples as reported previously [20]. Briefly, the samples were deparaffinized and hydrated and peroxidase activity was blocked with H_2_O_2_. Subsequently, antigen retrieval was performed using citrate or EDTA buffer. After application of Ultra V Block, the samples were incubated with NIS (1:100, NBP1-70342, Novus Biologicals, Centennial, CO, USA), CRYM (1:100, H00001428-M03, clone 6B3, Abnova, Taipeh, Taiwan), and THRB (1:100, 209-301-A96, Rockland Immunochemicals, Limerick, PA, USA) for 1 h at room temperature. Afterward, the tissue was incubated with the primary antibody enhancer and the horseradish peroxidase polymer according to the manufacturer’s protocol (UltraVision Plus Detection System, Thermo Scientific, Waltham, MA, USA). Staining was visualized with DAB Quanto Chromogen and Substrate (Thermo Scientific, CA, USA) or AEC (3-amino-9-ethylcarbazole) Substrate (BD Pharmingen™, San Diego, CA, USA). Staining was analyzed using an Olympus BH-2 microscope (Olympus, Tokyo, Japan) by the blinded researcher (L.K.W.) based on the intensity (0 = negative; 1 = weak; 2 = moderate; 3 = strong) and percentage (0–100%) of stained neoplastic cells. A score was calculated by multiplying staining intensity and percentage (0–300). Protein levels were considered negative when the formed score was <15.

### 2.3. Statistical Analysis

Statistical analysis was performed using Stata (Stata Corp., College Station, TX, USA) and Prism GraphPad software (GraphPad Software, San Diego, CA, USA). Categorical data were reported as absolute frequencies (%) and continuous parameters as median and interquartile ranges. Correlation analysis was performed using Fisher’s exact or chi-squared test. The overall survival (OS), CSS, or disease-free survival (DFS) was calculated from the date of diagnosis to the date of death, cancer-associated death, or recurrence, respectively. The median follow-up was calculated according to the method by Schemper and Smith [21]. Kaplan–Meier curves were used to visualize CSS and DFS curves and analyzed using the log-rank test. Univariable analysis was performed using the cox proportional hazard model. Multivariable analysis was not performed due to the low number of events (CSS: 20 events; DFS: 23 events) [22]. Interaction analysis was performed using a pairwise interaction model in the Cox regression analysis.

## 3. Results

### 3.1. Analysis at Baseline

In total, 44 patients with ACC of the head and neck were included in this study. Baseline characteristics are shown in Table 1. The cohort consisted of 41% (*n* = 18) men and 59% (*n* = 26) women. The median observation period was 121 months (49–199). The median age at the time of diagnosis was 60.5 years (47–70). Of the tumors, 34% (*n* = 15) were located in the major salivary glands and 66% (*n* = 29) were located in the minor salivary glands. Patients presented with an early-stage tumor (I-II) in 27% (*n* = 13) of the cases. Moreover, 23% (*n* = 10) of the patients showed lymph node metastasis and 7% (*n* = 3) of the patients had distant metastasis. Perineural invasion was found in 52% (*n* = 23) of the patients. Lymphovascular invasion was detected in 14% (*n* = 6) of the patients. Most patients were initially treated with surgery (84%; *n* = 37). Of these, 46% (*n* = 20) received postoperative radiotherapy and 21% (*n* = 9) received postoperative radio-chemotherapy. Disease recurrence was recorded in 52% (*n* = 23) of the cases. The calculated median OS was 98 months (35–163), the median CSS was 119 months (35–not reached), and the median DFS was 48 months (17–not reached).

### 3.2. Protein Levels of CRYM, THRB, and NIS and Correlation with Clinicopathological Features

**Figure 1:**
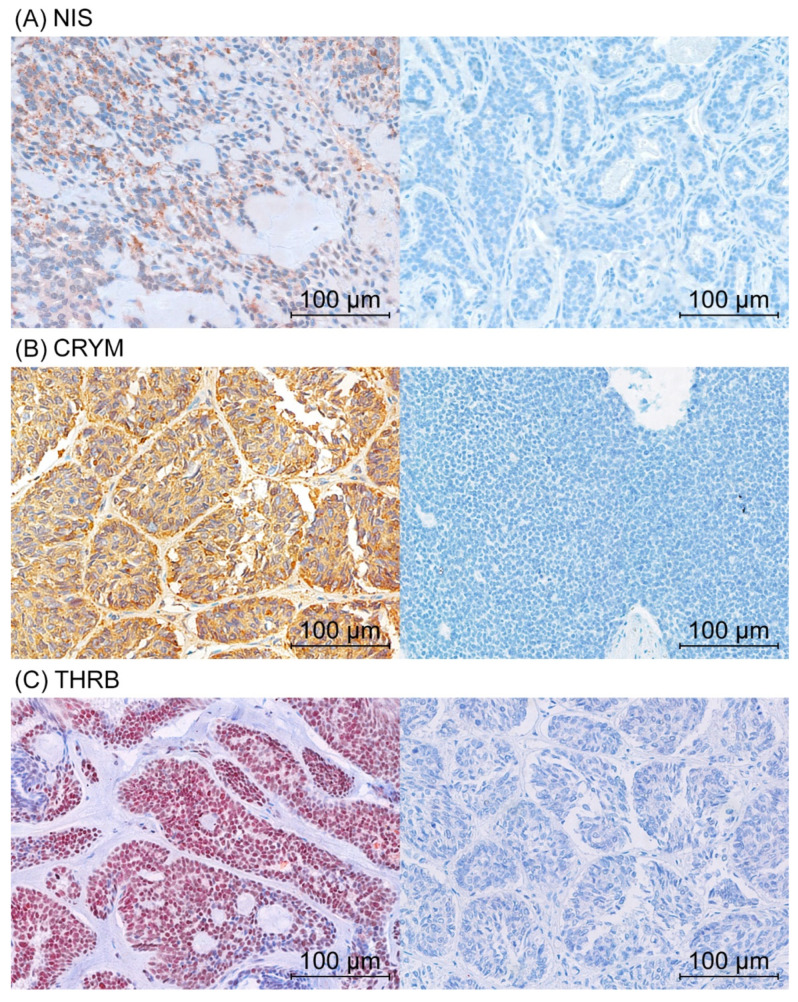
Immunohistochemical staining of NIS, CRYM, and THRB. Positive (**left**) and negative (**right**) immunohistochemical staining of NIS (**A**), CRYM (**B**), and THRB (**C**).

Protein levels were evaluated using immunohistochemistry (Figure 1). The protein levels of NIS, CRYM, and THRB were stratified into positive and negative levels, with the cutoff being a score < 15. NIS staining was mainly cytoplasmatic and positive in 72% (*n* = 31) of the cases. CRYM staining was generally cytoplasmatic and positive in 55% (*n* = 24) of the cases. THRB staining was nuclear and positive in 39% (*n* = 15) of the cases. Protein levels of CRYM, NIS, and THRB were not associated with each other. To further investigate correlation with clinicopathological features, we performed chi-squared or Fisher’s exact test. NIS protein was more commonly negative in patients older than 60 years (*p* = 0.015; corr. *p* = 0.045). After Bonferroni correction, there was no further correlation of NIS, CRYM, or THRB with staging, age, sex, grading, perineural, or lymphovascular invasion (Table S1). 

### 3.3. Analysis of Cause-Specific Survival and Disease-Free Survival 

**Figure 2:**
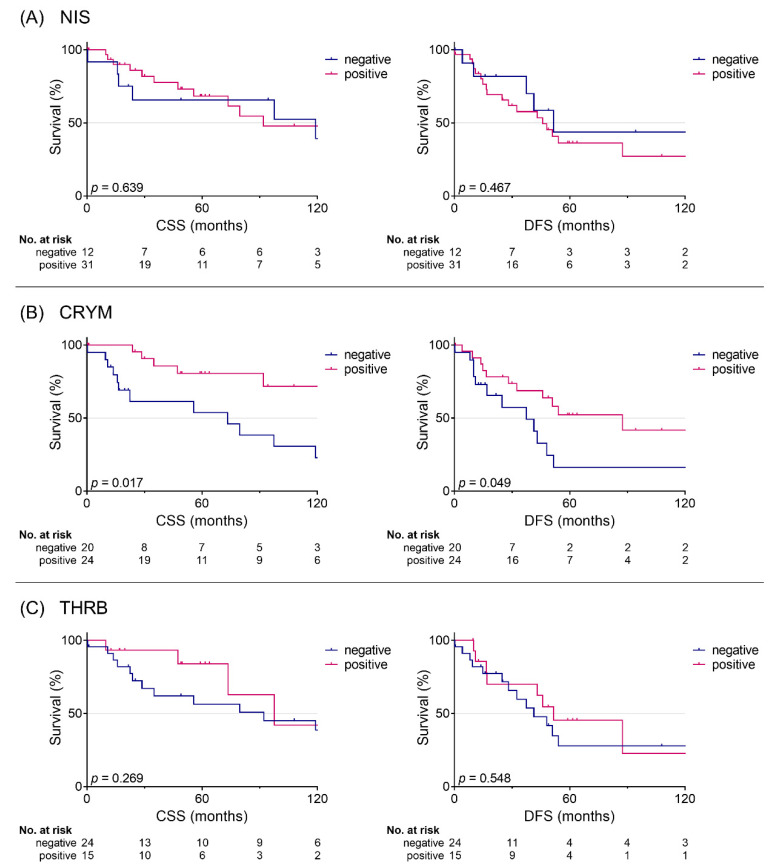
Kaplan–Meier survival curves. CSS and DFS for positive and negative protein levels of NIS (**A**), CRYM (**B**), and THRB (**C**). CSS: cause-specific survival; DFS: disease-free survival; *p*: log-rank *p*-value.

To analyze the prognostic value of NIS, CRYM, and THRB, we evaluated protein levels in relation to CSS and DFS (Figure 2; Table 2). Positive CRYM levels were associated with a better CSS (140 vs. 74 months; *p* = 0.017; corr. *p* = 0.051) and a better DFS (87 vs. 37 months; *p* = 0.049; corr. *p* = 0.147). Further univariable analysis revealed a significantly reduced risk of cancer-associated death in patients with positive levels of CRYM (HR = 0.34; 95% CI: 0.13–0.86; *p* = 0.023; corr. *p* = 0.069). However, the risk of recurrence was not affected in univariable analysis (HR = 0.44; 95% CI: 0.19–1.02; *p* = 0.054; corr. *p* = 0.162). NIS or THRB protein levels were not associated with DFS or CSS. 

## 4. Discussion

ACC is characterized by late recurrence and a poor long-term survival. To date, clinicopathological features, such as staging and perineural invasion, serve as prognostic markers. Surgery and radiotherapy are the main pillars of therapy since systemic therapeutic options show dismal effects. Therefore, new biomarkers and therapeutic targets are needed to stratify high-risk patients and improve therapeutic options, especially for recurrent or progressive disease [1,2,3]. In this study, we investigated the protein levels of NIS, CRYM, and THRB and their prognostic relevance in ACC of the head and neck.

NIS protein was present in most ACC patients (72%; *n* = 31). This finding is in accordance with the literature. Gainor et al. found positive NIS levels in 75% (*n* = 15) of ACC patients. However, due to the rarity of the disease, the size of the study cohort was limited and the association with prognosis was not evaluated [8]. In thyroid cancer, gastric cancer, and ovarian cancer, high NIS levels were associated with a worse prognosis [9,10,11,12]. Therefore, we investigated the prognostic relevance in ACC of the head and neck. In contrast to literature, NIS was not associated with CSS or DFS. In other cancer entities, NIS is expressed in varying degrees [23]. While radioiodine is an important pillar of thyroid cancer treatment, it is actively discussed in other cancer entities with high levels of NIS [5,11,23,24]. Since sialadenitis and xerostomia are common side effects of radioiodine treatment, together with the common expression of NIS, we hypothesize that radioiodine may be an interesting therapeutic option for NIS-positive ACC of the head and neck [6,7]. However, further investigation concerning the functionality of the expressed NIS protein is needed.

Next, CRYM protein levels were investigated. ACC patients showed positive CRYM levels in 55% (*n* = 24) of the cases. Positive protein levels were associated with a longer CSS (140 vs. 74 months; *p* = 0.017; corr. *p* = 0.051) and DFS (87 vs. 37 months; *p* = 0.049; corr. *p* = 0.147). Additionally, the risk of cancer-associated death (HR = 0.34; 95% CI: 0.13–0.86; *p* = 0.023; corr. p = 0.069) was decreased. However, univariable analysis did not reveal a decreased risk of recurrence. In prostate cancer, high levels of CRYM are associated with a better prognosis [14]. Mechanistically, CRYM binds T3 in the cytoplasm after binding of NADPH and thus leads to increased intracellular T3 levels. After dissociation of NADPH, T3 is released again and able to act via nuclear signaling [13]. Thyroid hormones have been linked to promotion of proliferation and progression of several cancers [4]. Although intracellular T3 levels are increased in the case of high CRYM levels, it was suggested that CRYM buffers the growth-promoting effect of T3 [14]. Therefore, we hypothesize that CRYM also mitigates the cancer-promoting effect of T3 in ACC of the head and neck. To date, however, there is no report on the effect of thyroid hormones in ACC. Thus, further investigation is warranted.

In this study, THRB protein was present in 38% (*n* = 15) of ACC patients. No associations of THRB levels with prognosis or clinicopathological parameters were found in ACC of the head and neck. Similarly, 38% of breast cancer patients showed high protein levels of THRB. However, in contrast to our results, high levels of THRB were associated with a longer CSS and a significantly reduced risk of cancer-associated death [18,19]. 

The limitations of this study are the retrospective design and the small study population due to the rarity of the disease. Furthermore, ACC is characterized by late recurrences, which might not be recorded during the median observation time of 121 months [3]. Multivariable analysis was not performed due to the low number of events. Therefore, the influence of confounders could not be investigated. Protein levels were investigated via immunohistochemical staining in formalin-fixed paraffin-embedded tissue. Although antigen retrieval was performed, some protein might have been lost in comparison to fresh tissue samples. However, due to strictly standardized procedures, we assume that the staining intensities are valid. Altogether, external validation of our results is recommended.

## 5. Conclusions

This is the first study to report CRYM as a prognostic marker for a better outcome in ACC of the head and neck. Furthermore, we validated that the majority of ACC patients express NIS. THRB is expressed in 38% of the patients; however, no association with outcome was found. In conclusion, these data warrant further investigation of NIS as a therapeutic target and CRYM as a prognostic marker. Moreover, investigation of the functionality of the NIS protein in ACC of the head and neck is recommended to evaluate the feasibility of radioiodine treatment.

## Figures and Tables

**Table 1:** Baseline patient characteristics of adenoid cystic carcinoma patients.

	Number of Patients	Percentage (%)
**Sex**		
Female	26	59%
Male	18	41%
**T Stage**		
1	6	14%
2	6	14%
3	8	18%
4	24	55%
**N Stage**		
0	34	77%
1	4	9%
2	6	14%
**M Stage**		
0	41	93%
1	3	7%
**Stage**		
I	5	11%
II	7	15%
III	4	9%
IV	27	59%
x	3	7%
**Grading-Spiro**		
1	29	66%
2	13	30%
3	2	5%
**Grading-Perzin/Szanto**		
1	10	23%
2	25	57%
3	9	20%
**Localization**		
Minor	15	34%
Major	29	66%
**Perineural Invasion**		
Yes	23	52%
No	21	48%
**Lymphovascular Invasion**		
Yes	38	86%
No	6	14%

**Table 2:** Univariable analysis of CSS and DFS and protein levels of NIS, CRYM, and THRB.

	Univariable		
	HR	95% CI	*p*-Value
**CSS**			
NIS	0.08	0.31–2.06	0.645
CRYM	0.34	0.13–0.86	0.023
THRB	0.54	0.17–1.65	0.276
**DFS**			
NIS	1.37	0.51–3.75	0.530
CRYM	0.44	0.19–1.02	0.054
THRB	0.76	0.32–1.85	0.549

HR: hazard ratio; CI: confidence interval; CSS: cause-specific survival; DFS: disease-free survival.

## Data Availability

The datasets of this study are available from the corresponding author on reasonable request.

## Supplementary Materials

The following are available online at https://www.mdpi.com/article/10.3390/jpm11121352/s1: Table S1: Correlation Analysis for Associations between Protein Levels of NIS, CRYM and THRB and Clinicopathological Features. Correlation was analyzed using Fisher’s exact test or chi-squared test.
