# Supplementary material for: Prognostic Relevance of Thyroid-Hormone-Associated Proteins in Adenoid Cystic Carcinoma of the Head and Neck
Source: J Pers Med. 2021 Dec 12;11(12):1352. doi: 10.3390/jpm11121352 (PMC8703850; doi:10.3390/jpm11121352)
Supplement: Supplementary file 1 [file jpm-11-01352-s001.zip › jpm-1474066-supplementary.pdf]

Table S1. Correlation analysis for associations between protein levels of NIS, CRYM, and THRB and clinicopathological features. Correlation was analyzed using Fisher's exact test or chi-squared test.

	NIS			CRYM			THRB		
	**Negative**	**Positive**	***p*-Value**	**Negative**	**Positive**	***p*-Value**	**Negative**	**Positive**	***p*-Value**
**Gender**									
Female	8 (67%)	18 (58%)		9 (45%)	17 (71%)		17 (71%)	6 (40%)	
Male	4 (33%)	13 (42%)	0.735	11 (55%)	7 (29%)	0.083	7 (29%)	9 (60%)	0.057
**Age**									
<60	2 (17%)	18 (58%)		9 (45%)	12 (50%)		11 (46%)	7 (47%)	
≥60	10 (83%)	13 (42%)	0.015	11 (55%)	12 (50%)	0.741	13 (54%)	8 (53%)	0.959
**T Stage**									
T1-2	2 (17%)	10 (32%)		5 (25%)	7 (29%)		4 (17%)	7 (47%)	
T3-4	10 (83%)	21 (68%)	0.456	15 (75%)	17 (71%)	0.757	20 (83%)	8 (53%)	0.043
**N Stage**									
N0	9 (75%)	24 (77%)		14 (70%)	20 (83%)		18 (75%)	11 (73%)	
N1-2	3 (25%)	7 (23%)	1.000	6 (30%)	4 (17%)	0.293	6 (25%)	4 (27%)	0.908
**M Stage**									
M0	11 (92%)	29 (94%)		18 (90%)	23 (96%)		22 (92%)	14 (93%)	
M1	1 (8%)	2 (6%)	1.000	2 (10%)	1 (4%)	0.430	2 (8%)	1 (7%)	1.000
**Stage**									
I–II	2 (17%)	10 (36%)		5 (26%)	7 (32%)		5 (23%)	6 (43%)	
III–IV	10 (83%)	18 (64%)	0.285	14 (74%)	15 (68%)	0.699	17 (77%)	8 (57%)	0.201
**Grading-Spiro**									
1	7 (58%)	21 (68%)		13 (65%)	16 (67%)		16 (67%)	11 (73%)	
2	4 (33%)	9 (29%)		5 (25%)	8 (33%)		7 (29%)	3 (20%)	
3	1 (8%)	1 (3%)	0.610	2 (10%)	0 (0%)	0.291	1 (4%)	1 (7%)	0.859
**Grading-Perzin/Szanto**									
1	4 (33%)	6 (19%)		5 (25%)	5 (21%)		6 (25%)	3 (20%)	
2	5 (42%)	19 (61%)		9 (45%)	16 (67%)		13 (54%)	9 (60%)	
3	3 (25%)	6 (19%)	0.483	6 (30%)	3 (13%)	0.277	5 (21%)	3 (20%)	1.000
**Perineural Invasion**									
No	7 (58%)	15 (48%)		12 (60%)	11 (46%)		14 (58%)	5 (33%)	
Yes	5 (42%)	16 (52%)	0.558	8 (40%)	13 (54%)	0.349	10 (42%)	10 (67%)	0.129
**Lymphatic Invasion**									
No	10 (83%)	27 (87%)		17 (85%)	21 (88%)		20 (83%)	14 (93%)	
Yes	2 (17%)	4 (13%)	1.000	3 (15%)	3 (13%)	1.000	4 (17%)	1 (7%)	0.631
